# Round about the Asylums. VII

**Published:** 1888-06-16

**Authors:** 


### ROUND ABOUT THE ASYLUMS.—VII.

BY A MEDICAL SUPERINTENDENT.

### JOINT COUNTIES ASYLUM, ABERGAVENNY.

THIS asylum takes the pauper lunatics from the counties of Merioneth, Brecon, and Radnor. Seven hundred and ninety-one patients were resident at the end of the year, and there are 70 vacant beds, being sufficient to meet the probable increase on the male side for five years, and on the female side for eleven years. 714 of the patients are chargeable to the three counties, 36 are private patients, and 29 are out-county cases, chiefly from Denbigh. There were 178 admissions, 118 discharges, and 56 deaths. The recoveries were 35·8 per cent. on the admissions; the deaths 7·3 per cent. on the average number resident. The weekly cost is 7*s.* 3*d.*, being a trifle higher than that for 1886. Upwards of 450 patients are employed in the usual asylum occupations, and nearly 600 are allowed to walk beyond the grounds. The library is well kept up by an annual grant of £20, and the associated entertainments are numerous. The Commissioners say, "according to the medical records, there has been great immunity from serious casualties, and complete freedom from exceptional or epidemic disorders." Dr. Glendinning shows that the curableness of insanity is greatly influenced by the duration of the disease previous to the adoption of treatment, and he reiterates the advantage to be derived from early sending patients to the asylum; but he "regrets to say the advantage is, in the majority of cases, ignored." It is most unfortunate that this advice, so frequently given by alienist physicians, is so rarely acted upon.

### BUCKS COUNTY ASYLUM.

At thirty-five years of age an asylum begins to get old; but this one shows a fair amount of vigour, although it this year issues its thirty-fifth annual report. There are 435 patients resident. Forty-four of these are out-county cases, and 15 are private patients. In addition to the beds occupied by these, there are some vacant ones available for the county, so that it is to be congratulated on having sufficient accommodation for its wants, present and prospective. One hundred and fifteen patients were admitted during the year, and the recoveries stand at 36 per cent. on the admissions. The deaths were 41, being a percentage of 9 on the average number resident. The weekly rate is 9*s.* 4*d.* We learn that there are in the asylum 59 idiots, 5 paralytics, and 67 epileptics—not a very promising lot out of a total of 435. The Commissioners remark that the tidiness of the patients is creditable to those in charge of them, and that the clothing is good. They also say that the appearance of the attendants is satisfactory, but that they are weak numerically. This can only refer to the day attendants, as the night staff would seem to be ample. There were very few minor casualties, and no serious one in the asylum; but two patients committed suicide when absent on probation. One of these had never shown any suicidal tendency, and the other was believed to be quite recovered. Such cases show the extreme difficulty, not to say impossibility, of judging of a patient's true mental state. Had either of these men been kept in the asylum there cannot be a doubt that many people would have declaimed against the injustice of "detaining sane people"; and had the new Lunacy Bill been in force, perhaps two general practitioners who knew nothing practically of insanity would have certified that they might be at large. The point is, that when such accidents happen with the expert who sees his patients daily, what can be looked for from the ordinary medical man who is to see the patient twice only? We are pleased to note that the farm accounts are properly kept, not only rent of land being charged, but also patients' labour and rates. As a consequence there was a loss of £115. Supposing the above-named items had been omitted, as in so many asylums, there would have been a profit of £60.

### BARNWOOD HOUSE ASYLUM, GLOUCESTER.

It is hard to know what to say about this and kindred institutions. Ought they to be praised for the success which has attended their efforts to obtain patients paying high rates, and for the efficient manner in which the most of them are conducted? Or ought they to be blamed for not being wholly charitable hospitals, and for not opening their doors to the really deserving poor, meaning by "poor" those who have seen better days, and whose only haven is too often the wards of a county asylum. Not being imperial institutions, ought they or ought they not to draw patients from private asylums? To discuss these questions would need time and space greater than our careful editor would allow, and such a discussion would, moreover, draw us far into the realms of controversy where assuredly we have no wish to enter. The poet has warned us that "fools rush in where angels fear to tread," and we heed the warning. Apart from conflicting views, there is a well-founded opinion that Barnwood House is one of the very best of its class. It can accommodate 154 patients, and it would seem to be nearly always full. The cost per head per week is 38*s.* 11*d.* We are pleased to note that ten patients are received gratuitously, and seventy-one at "reduced rates"; but this does not tell us much, as it may merely mean at a rate less than 38*s.* 11*d.*, and many private asylums admit patients for less than £2. What we could really extract information from would be a table showing the numbers paying less than 5*s.*, 10*s.*, and 20*s.* a-week respectively. The recoveries for last year were 45 per cent. on the admissions. The death-rate was only 1·9 per cent. on the average number resident. This is so surprisingly low that one would like to know whether paralytics, epileptics, and such cases are admitted or excluded. The committee do not seem to like the idea of paying income tax, and cases have been drawn up for a legal decision. Dr. Needham's patients seem to have written upwards of nine thousand letters during the year. As the demented and acute cases rarely write at all, there must be a good many Mr. Micawbers among the other classes. The medical wants of the 150 patients are seen to by Dr. Needham and two qualified assistants, and as the drug bill amounts to £80, they should be well attended to and well physicked.

